# “Always online”: How and when task interdependence and dispositional workplace anxiety affect workplace telepressure after hours

**DOI:** 10.1002/pchj.747

**Published:** 2024-03-26

**Authors:** Xiaoyan He, Qin Gao, Ya Cao, Ran Bian, Xiao‐Hua (Frank) Wang

**Affiliations:** ^1^ Beijing Key Laboratory of Applied Experimental Psychology, National Demonstration Center for Experimental Psychology Education, National Virtual Simulation Center for Experimental Psychology Education, Faculty of Psychology Beijing Normal University Beijing China; ^2^ School of Sociology China University of Political Science and Law Beijing China

**Keywords:** dispositional workplace anxiety, others' approval contingency of self‐worth, perception of pay‐for‐responsiveness, task interdependence, workplace telepressure after hours

## Abstract

Information and communication technology (ICT) provides employees with convenience in communication. However, it also creates a preoccupation with and urges to respond quickly to work‐related ICT messages during nonworking time, which is defined as workplace telepressure after hours (WTA). Drawing on the job demand–resource model, conservation of resource theory, and workplace anxiety theory, this study explores how and when task interdependence and dispositional workplace anxiety affect WTA and how individuals cope with WTA. A total of 269 full‐time workers from an online survey panel completed questionnaires at three time‐points. We found that both task interdependence and dispositional workplace anxiety are positively related to WTA. The perception of pay‐for‐responsiveness moderates the relationship between task interdependence and WTA, such that the relationship is significant only for employees with a strong perception of pay‐for‐responsiveness. Others' approval contingency of self‐worth moderates the relationship between dispositional workplace anxiety and WTA, and the relationship is significant only for employees with high degrees of others' approval contingency of self‐worth. Finally, WTA arising from external work requirements or the internal pursuit of achieving work goals prompts employees to generate responsiveness coping strategies. Overall, these findings suggest that task interdependence and dispositional workplace anxiety are important factors affecting employees' WTA and highlight the importance of being responsive to WTA.

## INTRODUCTION

The widespread use of information and communication technologies (ICTs), such as cell phones and computers, for maintaining communication with employees has become a common practice (Nagar & Talwar, 2021; Y. Park et al., 2020). ICTs facilitate the seamless sharing of information, and enhance communication and collaboration among employees (Day et al., 2010; Diaz et al., 2012). Despite the benefits of using ICTs, the practice also leads to many negative outcomes. Specifically, it blurs the boundaries between working and nonworking time, resulting in reduced psychological detachment from work (Charalampous et al., 2019; Derks et al., 2014; Y. Park et al., 2011). Consequently, employees face challenges in detaching from work, hampering their ability to recover from work. This, in turn, exacerbates work–family conflict (Day et al., 2019; Derks et al., 2015; Harris et al., 2015), reduces work–life balance (Carvalho et al., 2015), and contributes to insomnia and depression (Panova & Lleras, 2016; Thomée et al., 2007). Notably, research highlights that the adverse outcomes associated with ICTs are closely linked to workplace telepressure (Barber & Santuzzi, 2015, 2017; Kotera et al., 2021).

Workplace telepressure, as first conceptualized by Barber and Santuzzi (2015), refers to the urge to respond quickly to work‐related ICT information. It represents a psychological state in which employees feel compelled to maintain a constant connection with their work activities. According to Barber and Santuzzi (2015), workplace telepressure is driven by the demands of the job. To meet the organization's expectations of being continuously “online,” employees have to always focus on their work (Boswell & Olson‐Buchanan, 2007; McMillan & Shockley, 2019), leading to an increase in workplace telepressure (Barber et al., 2019; Barber & Santuzzi, 2015, 2017; Hu et al., 2019). Specifically, employees who face high job demands, such as techno‐overload and response expectations, experience heightened levels of telepressure (Barber & Santuzzi, 2015). Moreover, further studies have shown that workplace telepressure is influenced not only by job demands but also by personality traits such as self‐control and fear of missing out (Barber & Santuzzi, 2017). Besides general personality traits (e.g., self‐control and neuroticism), the work‐related personality trait of workaholism has also been found to be positively associated with workplace telepressure (Grawitch et al., 2018).

Although prior research has offered valuable insights into the factors contributing to workplace telepressure, limitations still exist. First, these studies have not differentiated between telepressure during working and nonworking hours. For example, the measurement items for workplace telepressure do not specify the time of day (Barber & Santuzzi, 2015, 2017; Grawitch et al., 2018), as seen in items such as “It is hard for me to focus on other things when I receive a message from someone” and “I have an overwhelming feeling to respond right at that moment when I receive a request from someone.” These items mainly emphasize an individual's perception of pressure to focus on work‐related information upon receiving it. However, it is essential to recognize that the factors contributing to telepressure may differ between working hours and nonworking hours. Employees’ practice of responding to work‐related messages promptly during working hours is within the scope of their work responsibilities. Therefore, responses to work‐related messages during working hours are generally accepted and understood. Yet, responding to work‐related ICT messages during nonworking hours may be perceived as an additional workload and disruption. The factors contributing to telepressure during nonworking hours may typically result from external work‐related demands (i.e., the demands that are driven by the work environment; Giurge & Woolley, 2022; Grawitch et al., 2018) or intrinsic motivation (Giurge & Woolley, 2022). Crucially, workplace telepressure is linked to a series of adverse outcomes, such as reduced employee recovery resources (Barber et al., 2019) and sleep quality (Barber & Santuzzi, 2015, 2017; Hu et al., 2019), disrupted work–life balance (Barber et al., 2019; Barber & Santuzzi, 2017), and increased work–family conflict (Barber et al., 2019; Kao et al., 2020), mainly occurring in the family domain. Hence, it is crucial to investigate telepressure during nonworking hours separately and understand the reasons why employees feel an urgent need to respond to work‐related information during nonworking hours.

Second, the existing research highlights the influence of job demands and personality traits on telepressure. However, an essential aspect that warrants attention is that these studies lack consideration for the boundary effects shaping these impacts. Moreover, these studies only consider the impact of ICT‐related job demands or workplace norms on telepressure, such as ICT demands (Grawitch et al., 2018; Kao et al., 2020) and response norms (Barber & Santuzzi, 2015); no study has yet considered the potential impact of workplace demands that are not related to ICT. Third, while studies have unveiled the adverse outcomes linked to telepressure, there exists a gap in the literature regarding the proactive coping employed by employees in response to this phenomenon. It is imperative to delve into the strategies that individuals use to navigate and mitigate the detrimental effects of telepressure, paving the way for a more holistic understanding of how employees cope with the challenges posed by constant connectivity in the modern workplace.

**FIGURE 1:**
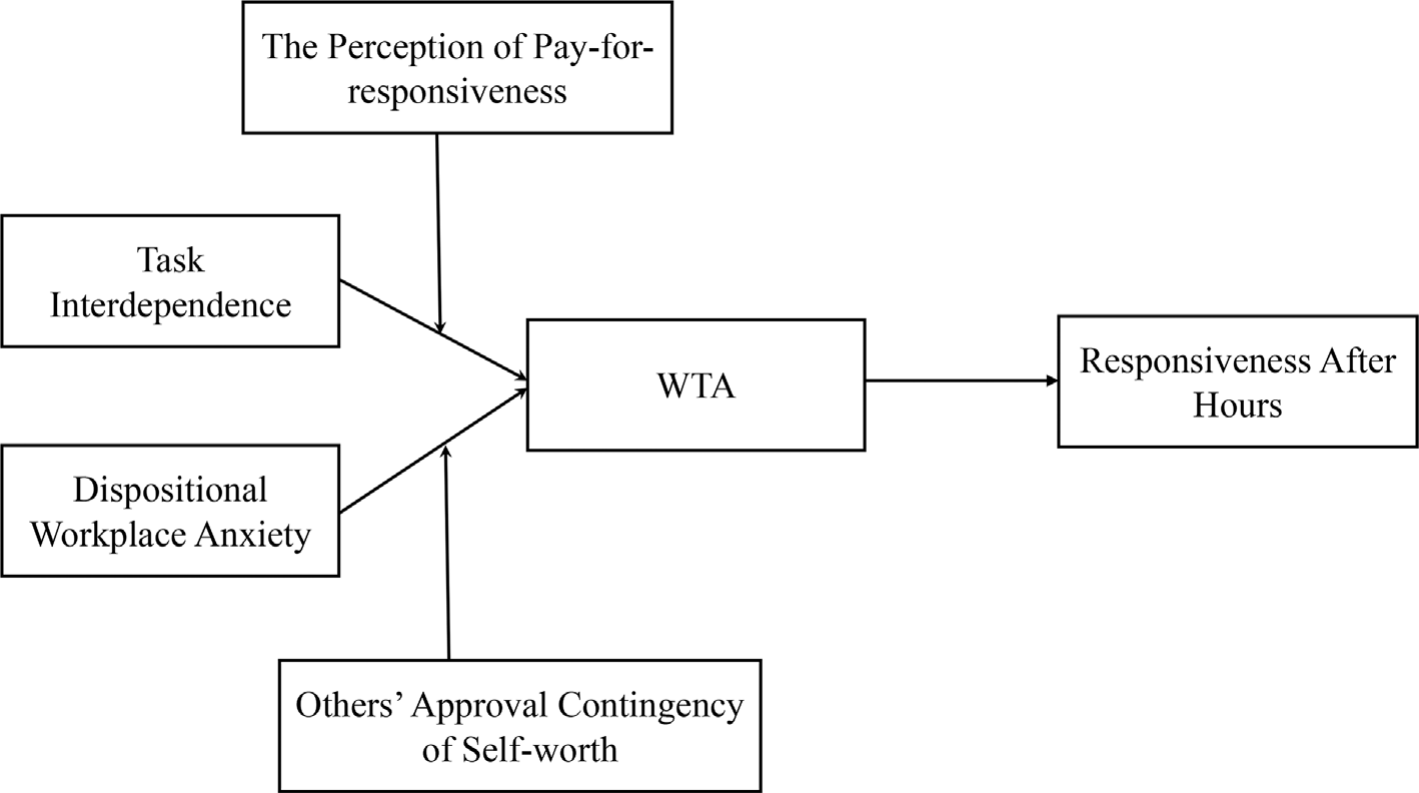
Hypothesized research model. WTA = workplace telepressure after hours.

Thus, to address these limitations and gain a comprehensive understanding of why employees feel an urgent need to respond to work‐related information during nonworking hours and how they cope with telepressure, this study focuses on workplace telepressure after hours (WTA). We aim to explore the factors influencing WTA, which include both personality traits and job demands that do not relate to ICT. Building on Barber and Santuzzi (2015), we define WTA as the experience of feeling compelled to respond to work‐related information during nonworking hours. Drawing from the job demands–resources model and conservation of resource (COR) theory, we propose that task interdependence (i.e., the external job demands that are driven by the work environment; Kiggundu, 1983) will be positively associated with WTA, and that the perception of pay‐for‐responsiveness will moderate this relationship. According to workplace anxiety theory, we propose that dispositional workplace anxiety (i.e., personality traits) will positively relate to WTA, and that others' approval contingency of self‐worth will moderate this relationship. Additionally, according to COR theory, we propose that when employees find themselves in a WTA state where their resources may face depletion, they may adopt a proactive coping strategy to deal with the situation. That is, WTA may trigger employees' responsiveness to overcome this state. Our hypothesized model is presented in Figure 1.

### Theoretical background and hypothesis development

#### Task interdependence and WTA: Perception of Pay‐for‐Responsiveness as a moderator

Task interdependence, defined as the extent to which employees rely on others to complete their work, reflects the close connections between individuals in the workplace (Kiggundu, 1983; Morgeson & Humphrey, 2006; Pearce & Gregersen, 1991). In situations of high task interdependence, employees find it challenging to independently accomplish work‐related tasks solely based on their own information resources and efforts (Morgeson & Humphrey, 2006). This high level of task interdependence fosters interactive and collaborative behaviors among organizational employees, compelling them to engage in frequent interpersonal interactions to fulfill work‐related responsibilities (Chong et al., 2020; Cleavenger et al., 2007; Morgeson & Humphrey, 2006). Thus, task interdependence is always viewed as an job demand(Kiggundu, 1983; Van Laethem et al., 2018).

According to the job demands–resources model, the characteristics of a job can be categorized into job demands and job resources (Demerouti et al., 2001). Job demands involve aspects of the job requiring physical, psychological, or emotional effort, leading to specific costs or strains for the employee. Conversely, job resources are factors within the work environment that support employees in achieving their goals (Demerouti & Bakker, 2011). Task interdependence is recognized as a job demand that depletes employees' physical and mental resources (Bakker & Demerouti, 2007; Kiggundu, 1983; Parker, 2014). In the era of ICTs, the impact of task interdependence on employee resource depletion is accentuated, as ICT renders the frequent interpersonal interaction required by task interdependence easily accessible at any time and anywhere, even after working hours (Cho et al., 2020; Y. Park et al., 2020). Additionally, empirical evidence shows that task interdependence is positively related to exhaustion (Chong et al., 2020). Specifically, when employees fail to complete their work promptly, it affects the progress of the entire interdependent employee group or team. This, in turn, increases the employee's psychological burden, such as increasing feelings of guilt which are positively related to emotional exhaustion (Chong et al., 2020; Liden et al., 1997; Thompson, 2003).

Based on COR theory, individuals are motivated to accumulate, safeguard, and cultivate their self‐resources. When their resources are threatened with loss, they are motivated to protect their resources (Hobfoll et al., 2018; Xia et al., 2019) and to avoid suffering more negative outcomes (Halbesleben et al., 2014). Consequently, individuals become concerned about an event that leads to resource depletion and strive to manage it (Halbesleben & Bowler, 2007). Therefore, we propose that when faced with the requirements of task interdependence, employees will want to fulfill the external requirements of their work to avoid a further loss of resources with serious consequences, thereby prompting them to experience a psychological urge to respond to work‐related ICT information quickly. Hence, we propose the following hypothesis.

Hypothesis 1 Task interdependence is positively related to WTA.

Further, we propose that the perception of pay‐for‐responsiveness moderates the relationship between task interdependence and WTA. The perception of pay‐for‐responsiveness is defined by the employee's perceived strength of the relationship between an immediate response to work‐related information and the payment they receive. The conceptualization of the perception of pay‐for‐responsiveness is rooted in the definition of the perception of pay‐for‐performance, which involves an employee's assessment of the strength of the relationship between their performance and the pay they receive (Heneman, 1992; Heneman et al., 1988; Zhang et al., 2015). In organizations, the prompt response to work‐related messages is pertinent to the accomplishment of work‐related tasks. Thus, employees' performance in responding to work‐related messages can be considered an indicator of job performance (Cheng & McCarthy, 2018). Therefore, we specify the pay‐for‐performance to the pay‐for‐responsiveness.

Prior research on the perception of pay‐for‐performance indicates that pay‐for‐performance functions as a significant work stressor, requiring employees to achieve a certain level of work‐related performance (Kong et al., 2022; Park & Sturman, 2021). Thus, employees always perceive pay‐for‐performance as a threat that compels them to meet the organization's performance expectations. They fear that failing to meet performance expectations could lead to undesirable consequences, such as receiving lower salary increases compared with their colleagues or even facing employment termination (Kong et al., 2022). In alignment with the perception of pay‐for‐performance, pay‐for‐responsiveness is also considered a stressor, as the widespread use of ICT creates expectations that individuals are obligated to respond to work‐related messages promptly (Day et al., 2012). The failure to respond promptly to work‐related messages may lead to the formation of a negative work image in the eyes of superiors or colleagues (Barber & Santuzzi, 2017). As a result, this has the potential to adversely impact the employee's performance evaluation. Owing to employees' uncertainty about the association between pay and responsiveness, they may be concerned that their failure to respond promptly to messages could have a detrimental effect on their performance, thereby increasing their burden and resource depletion (Kong et al., 2022; Smit & Montag‐Smit, 2019). Therefore, we propose that perception of pay‐for‐responsiveness may exacerbate the resource depletion caused by task interdependence, leading to stronger feelings of WTA. We propose the following hypothesis:

Hypothesis 2 The perception of pay‐for‐responsiveness moderates the relationship between task interdependence and WTA, and this relationship is stronger when the perception of pay‐for‐responsiveness is high.

#### Dispositional workplace anxiety and WTA: Others' approval contingency of self‐worth as a moderator

We propose that dispositional workplace anxiety is an important individual difference (i.e., the personality traits that differ between individuals) related to WTA. Dispositional workplace anxiety is defined as the personality traits in feelings of anxiety, unease, and tension about job performance (Cheng & McCarthy, 2018; McCarthy et al., 2016). It represents a specific situational characteristic associated with work (Jex, 1998). According to the theory of workplace anxiety, dispositional workplace anxiety can facilitate job performance (Cheng & McCarthy, 2018; Mughal et al., 1996). When anxious individuals feel the difference between the expected work goal progress and the actual goal progress, a sense of anxiety arises. This anxiety drives individuals to reflection and self‐regulation, and increases their effort or engagement in work, thus leading to better performance (Cheng & McCarthy, 2018). Moreover, anxious employees tend to be deliberate and goal‐oriented (Carver et al., 2008). They will carefully examine external information and predict the consequences of their actions before making decisions, which helps them achieve their objectives (Carver & Scheier, 2011). Furthermore, anxious employees tend to invest more effort and strategic planning to reach their goals and avoid negative outcomes (Maner et al., 2007; Norem & Chang, 2002). Receiving work‐related messages during nonworking hours often suggests that work task goals are not completed (Richardson & Benbunan‐Fich, 2011). Thus, we suggest that employees with dispositional workplace anxiety may be more likely to pay attention to work‐related messages and experience stronger feelings of WTA. We propose the following hypothesis:

Hypothesis 3 Dispositional workplace anxiety is positively related to WTA.

Furthermore, we posit that others' approval contingency of self‐worth will moderate the relationship between dispositional workplace anxiety and WTA. Others' approval contingency of self‐worth refers to an individual's evaluation of their own value and self‐esteem being contingent upon the approval of and acknowledgment from others (Crocker, Karpinski, et al., 2003; Crocker, Luhtanen, et al., 2003). In this context, personal self‐worth and self‐esteem are shaped more by external factors than by internal self‐perception. Individuals with high levels of others' approval contingency become excessively reliant on external opinions and judgments to ascertain their own value (Crocker et al., 2002). Furthermore, the self‐worth of such individuals is predominantly tied to meeting goals set by others (Crocker & Knight, 2005; Crocker & Wolfe, 2001; Horberg & Chen, 2010; Prieler et al., 2021). For those with a high degree of others' approval contingency of self‐worth, meeting the expectations of others becomes a vital source of self‐worth (Horberg & Chen, 2010). When they succeed in meeting others' expectations, their self‐esteem escalates; conversely, it diminishes if they fail to do so (Crocker & Wolfe, 2001).

In the context of workplace communication, when employees receive work‐related messages from their coworkers or superiors, it signals an expectation for them to engage with the content and respond, implying the presence of tasks to be completed (Y. Park et al., 2020). Consequently, employees characterized by dispositional workplace anxiety and a high degree of others' approval contingency of self‐worth may feel a heightened urgency to respond promptly to such messages. Conversely, anxious employees with lower levels of others' approval contingency of self‐worth may be less concerned about external opinions (Crocker & Knight, 2005), with their response to messages driven primarily by the motivation to complete tasks rather than to obtain self‐worth validation. Therefore, we hypothesize that the impact of dispositional workplace anxiety on WTA will be more pronounced for employees with higher levels of others' approval contingency of self‐worth. Accordingly, we propose the following hypothesis:

Hypothesis 4 Others' approval contingency of self‐worth moderates the relationship between dispositional workplace anxiety and WTA; this relationship is stronger when others' approval contingency of self‐worth is higher.

#### WTA and responsiveness after hours

Responsiveness is the behavior by which employees respond quickly to work‐related messages during nonworking hours (Sonnentag et al., 2018). We propose that WTA will promote responsiveness after hours for two main reasons. First, research has shown that telepressure can lead employees to exhibit a heightened focus on work‐related information, continuously activating their thoughts related to work (Hu et al., 2019). This heightened telepressure leads individuals to frequently shift their attention toward work‐related information, resulting in a persistent difficulty in disengaging from work and continuous resource depletion (Gillet et al., 2023; Pfaffinger et al., 2020). According to COR theory, individuals facing resource loss tend to take actions to protect their remaining resources (Halbesleben et al., 2014). Second, employees with high telepressure may overestimate the expectations of those initiating work‐related information, leading to a perceived need for swift responses (Barber & Santuzzi, 2015). This overestimation may trigger even faster response behaviors, as evident in studies showing higher email response frequencies and quicker email response speeds among individuals facing high workplace telepressure (Barber & Santuzzi, 2015; Cambier & Vlerick, 2020; Van Laethem et al., 2018). Additionally, employees experiencing high telepressure are more likely to prioritize the processing of work‐related information, placing it at the forefront of their task priorities (Gupta et al., 2013; Kalman et al., 2013; Wajcman & Rose, 2011). Based on the above, individuals in a WTA state may be eager to promptly respond to messages, driven by the urgency to protect their resources and to meet other's expectations (Sonnentag et al., 2018). Accordingly, we propose the following hypothesis:

Hypothesis 5 WTA is positively related to responsiveness after hours.

Combined with the above assumptions, we propose the following hypotheses:

Hypothesis 6a Task interdependence has an indirect effect on responsiveness after hours via WTA.

Hypothesis 6b Dispositional workplace anxiety has an indirect effect on responsiveness after hours via WTA.

Hypothesis 7a The perception of pay‐for‐responsiveness moderates the indirect relationship between task interdependence and responsiveness after hours via WTA, such that the positive indirect relationship is stronger for employees with a higher perception of pay‐for‐responsiveness.

Hypothesis 7b Others' approval contingency of self‐worth moderates the indirect relationship between dispositional workplace anxiety and responsiveness after hours via WTA, such that the positive indirect relationship is stronger for employees with higher others' approval contingency of self‐worth.

## METHODS

### Participants and procedure

Participants were full‐time workers recruited through Credamo, a widely recognized and used online questionnaire platform (Q. Zhang et al., 2022). Participants were required to meet two basic requirements: (1) to use communication tools (e.g., cell phones, computers) and instant messaging software (e.g., WeChat, QQ, Lark, or DingTalk) at work; and (2) to be full‐time employees who have a regular commute to work. To ensure the quality of the participants, following previous studies (Q. Zhang et al., 2022), we first asked Credamo to send links only to full‐time employees in their database 1 week before the main study. Finally, a total of 333 participants were willing to participate in this study.

Participants completed questionnaires at three time‐points. At Time 1 (T1), participants completed measures of task interdependence, dispositional workplace anxiety, the perception of pay‐for‐responsiveness, and others' approval contingency of self‐worth. At Time 2 (T2), 1 week after T1, we collected participants' demographic information and WTA. At Time 3 (T3), 1 week after T2, participants completed the measurement of responsiveness after hours. The research was examined and approved by the Ethics Committee of Beijing Normal University, and we obtained informed consent from participants. According to the requirements of the Ethics Committee and the common practice of previous research (Barber et al., 2019; Grawitch et al., 2018), participants who completed all the surveys were compensated with 20 RMB ($3) for their time.

After three surveys, a total of 271 participants had completed all of the questionnaires. One participant was excluded for inconsistent demographic information, and one for an incorrect response to the attention check item (the item was “Please select ‘strongly disagree’ for this item”; participants who chose any other option were considered as not meeting the requirements), and thus the final number of participants was 269. Given that path analysis is an extension of multiple regression analysis (Streiner, 2005), we conducted a post hoc analysis based on linear multiple regression using G*Power software (Version 3.1; and can be accessed at http://www.psychologie.hhu.de/arbeitsgruppen/allgemeine‐psychologie‐und‐arbeispsycholo). We set a significance level of 0.05, a medium effect size of 0.15, and a sample size of 269. The results showed that our sample size exceeded the 95% level of statistical power, which suggests that our sample has good statistical testing power (Faul et al., 2009). Among the participants, 142 were male (52.8%), 127 were female (47.2%), 24.4% were unmarried, 75.3% were married, and 0.4% were divorced. The average age was 35.77 years (*SD* = 5.42), ranging from 20 to 55 years. In terms of education, 9.6% of the participants had a high school education, 14.0% had a college degree, 63.8% had a bachelor's degree, and 12.2% had a postgraduate degree. In terms of job levels, the participants' job positions were mainly concentrated at the lower (60.5%) and middle (33.9%) levels, with only a small number of participants having senior manager status (5.5%). The participants' occupations were in management (20.7%), technology research and development (20%), administration (10.7%), public services (9.2%), sales (8.9%), production (7%), finance (5.5%), and teaching (5.5%). 

### Measures

Unless otherwise noted, the response scales for all items ranged from 1 = *strongly disagree* to 5 = *strongly agree*.

#### Task interdependence

We used the Task Interdependence Scale with five items developed by Pearce and Gregersen (1991). An example item is “I work closely with others in doing my work” (α = .68).

#### Dispositional workplace anxiety

We measured dispositional workplace anxiety using the Performance Anxiety Scale with eight items (McCarthy et al., 2016). An example item is “I am overwhelmed by thoughts of doing poorly at work” (α = .94).

#### Perception of pay‐for‐responsiveness

We measured perception of pay‐for‐responsiveness by adapting a three‐item scale from Deckop et al. (1999). The original scale was used to measure the strength of the relationship between employees' perceptions of their performance and pay, and we adapted the general performance of the original items to the specific performance of “responding to work information.” An example item is “Being more effective in responding to work information means higher pay for me” (α = .83).

#### Others' approval contingency of self‐worth

We measured others' approval contingency of self‐worth using the subscale related to “others' approval” in the Contingencies of Self‐Worth Scale developed by Crocker et al. (2003), which includes five items. An example item is “What others think of me has no effect on what I think about myself” (α = .84).

#### Workplace Telepressure after hours (WTA)

WTA was measured with six items from the Workplace Telepresure Scale developed by Barber and Santuzzi (2015). Because Barber and Santuzzi's scale (2015) does not distinguish between working time and nonworking time workplace telepressure, our study was modified to emphasize receiving messages from work during nonworking time. An example item is “After work, it's hard for me to focus on other things when I receive a message from work” (α = .80).

#### Responsiveness after hours

Responsiveness after hours was measured with three items from the responsiveness scale developed by Sonnentag et al. (2018). Participants were asked to rate how often they engaged in certain behaviors when they received a work‐related message (e.g., from a supervisor, coworker, or client) during nonworking hours. An example item is “After work, I respond immediately to work messages when I receive them, even when I am busy with other things.” The response scales ranged from 1 = *hardly ever* to 5 = *almost always* (α = .80).

#### Controls

We controlled for demographics, including participants' age, sex, occupation, job level, education, and marital status, because of their potential effects on employee behavior (Gao et al., 2024; Ng & Feldman, 2008).

### Results

#### Preliminary analyses

Before testing the hypotheses, we conducted a series of confirmatory factor analyses of the measurement models that included the focal predictors (task interdependence and dispositional workplace anxiety), moderators (perception of pay‐for‐responsiveness and others' approval contingency of self‐worth), mediator (WTA), and outcome (responsiveness after hours) using Mplus 8.3 (Muthén & Muthén, 1998–2017) to determine whether our measured variables were distinguishable from each other. We evaluated several model fit indices, which include the comparative fit index (CFI), the Tucker‐Lewis index (TLI), the standardized root mean square residual (SRMR), and the root mean square error of approximation (RMSEA). The results show that the data fit the six‐factor model well [χ^2^(387) = 620.95, CFI = 0.94, TLI = 0.93, SRMR = 0.06, RMSEA = 0.05], and the model fit significantly better than the alternative models: (1) the five‐factor model, which combined WTA and responsiveness after hours into one factor (Δχ^2^ = 220.51, Δ*df* = 8, *p* < .001); (2) the four‐factor model, which combined WTA and responsiveness after hours into one factor, and combined dispositional workplace anxiety and others' approval contingency of self‐worth into one factor (Δχ^2^ = 518.74, Δ*df* = 12, *p* < .001); and (3) the three‐factor model, which combined WTA and responsiveness after hours into one factor, task interdependence and the perception of pay‐for‐responsiveness into one factor, and dispositional workplace anxiety and others' approval contingency of self‐worth into one factor (Δχ^2^ = 911.52, Δ*df* = 15, *p* < .001). These results support the discriminant validity of the measures.

#### Test of hypotheses

Descriptive statistics (i.e., means, *SD*s, reliability, and correlation coefficients) for the study variables are presented in Table 1. As shown in Table 1, task interdependence was positively related to both perception of pay‐for‐responsiveness (*r* = 0.25, *p* < .001) and WTA (*r* = 0.18, *p* = .001). Perception of pay‐for‐responsiveness also had a positive relationship with WTA (*r* = 0.15, *p* = .006). Dispositional workplace anxiety was positively related to both others' approval contingency of self‐worth (*r* = 0.57, *p* < .001) and WTA (*r* = 0.15, *p* = .006). Others' approval contingency of self‐worth was positively related to WTA (*r* = 0.14, *p* = .011). Moreover, WTA was positively related to responsiveness after hours (*r* = 0.48, *p* < .001). These results indicate that there was a significant correlation among the variables. Based on this, we conducted a model analysis to test our hypotheses.

**TABLE 1:** Descriptive statistics and correlations among the study variables.

	*M*	*SD*	1	2	3	4	5	6	7	8	9	10	11	12
1. Sex (T2)	0.47	0.50	–											
2. Age (T2)	35.77	5.43	−0.15**	–										
3. Occupation (T2)	7.40	3.28	0.05	−0.01	–									
4. Rank (T2)	1.45	0.60	−0.10	0.18**	0.17**	–								
5. Education (T2)	4.78	0.83	0.01	−0.15**	0.26*	0.12	–							
6. Marriage (T2)	1.76	0.45	−0.08	0.33**	0.07	0.08	0.05	–						
7. Task interdependence (T1)	3.86	0.56	−0.12*	−0.13*	0.04	0.08	0.10*	−0.07	(0.68)					
8. PPR (T1)	3.26	0.97	0.02	−0.05	−0.01	0.09	0.03	0.04	0.25**	(0.83)				
9. DWA (T1)	3.05	1.04	−0.02	0.06	−0.09	−0.02	0.01	−0.11*	−0.04	−0.06	(0.94)			
10. OACS (T1)	2.81	0.84	0.10	−0.10	−0.07	−0.03	0.03	−0.11*	0.07	0.03	0.57**	(0.84)		
11. WTA (T2)	3.90	0.62	0.10	0.04	0.01	0.06	−0.06	−0.01	0.18**	0.15**	0.15**	0.14*	(0.80)	
12. Responsiveness after hours (T3)	3.85	0.71	0.12*	0.16**	0.01	0.09	−0.11*	0.12*	0.05	0.09	−0.06	−0.02	0.48**	(0.80)

*Note*: *N* = 269. Coefficient alphas are reported along the diagonal in parentheses. Sex: 0 = male; 1 = female.

Abbreviations: DWA = dispositional workplace anxiety; OACS = others' approval contingency of self‐worth; PPR = perception of pay‐for‐responsiveness; WTA = workplace telepressure after hours.

* *p <* .05;

** *p <* .01, one‐tailed.

Following Wu et al. (2016), we tested our hypotheses in Mplus 8.3 using path analysis with two models. In addition to the hypothesized relations, we controlled for the direct effects of task interdependence and dispositional workplace anxiety on responsiveness after hours. We also controlled for sex, age, occupation, job level, education, and marital status by including paths from these factors to the mediators and outcome variables. Given that all hypotheses were directional, we employed one‐tailed tests, a commonly adopted practice in organizational behavior research (Bindl et al., 2019; Gao et al., 2024; Jones, 1954; Kimmel, 1957).

**FIGURE 2:**
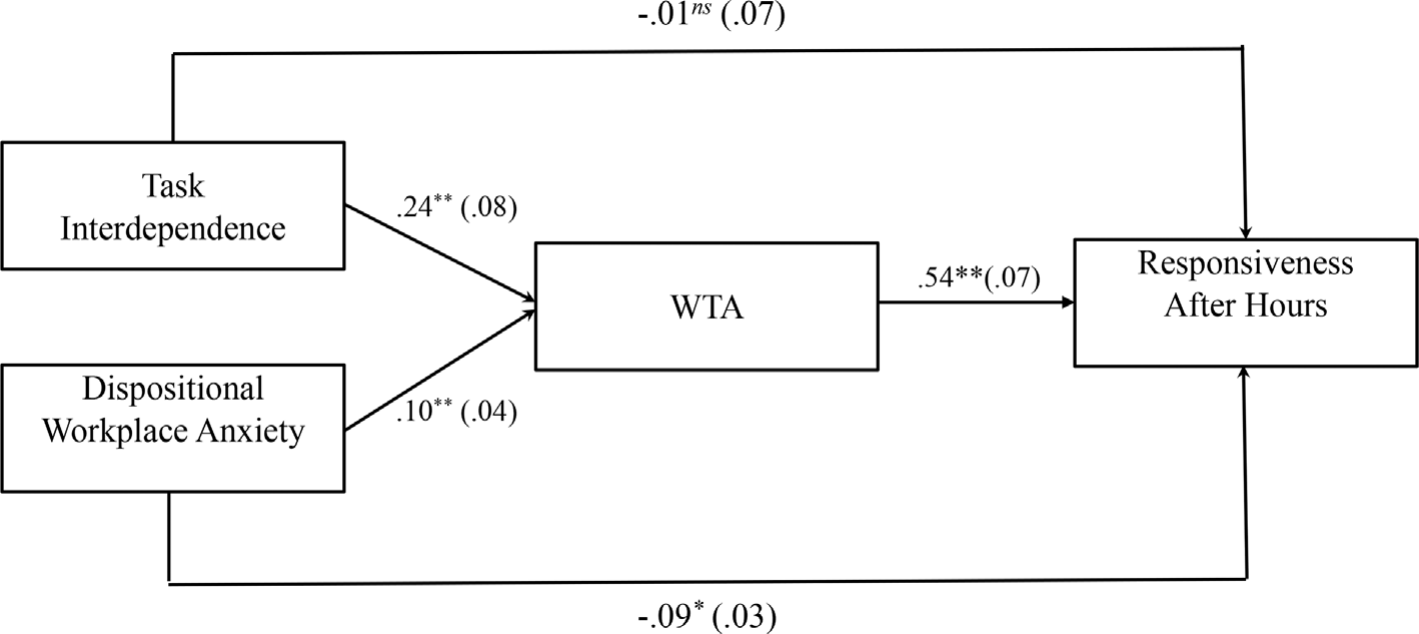
Unstandardized coefficients for the mediation‐only model (Model 1). WTA = workplace telepressure after hours. Path estimates are unstandardized regression coefficients. Standard errors are in parentheses. *p < .05, **p < .01, one‐tailed.

In the first model, both the direct effects and the mediating effects were considered. This model specification included the direct effects of task interdependence and dispositional workplace anxiety on WTA, the direct effects of WTA on responsiveness after hours, and the mediating effects of WTA between the two antecedent variables and responsiveness after hours. The first model fit showed that this is a saturated model, which has a perfect fit with zero degrees of freedom [*χ* ^*2*^(0) = 0, CFI = 1, TLI = 1, RMSEA = 0, SRMR = 0]. The results of the path analysis are displayed in Figure 2. The results show that task interdependence was positively and significantly related to WTA (*b* = 0.24, *SE* = 0.08, *p* < .001), dispositional workplace anxiety was positively and significantly associated with WTA (*b* = 0.10, *SE* = 0.04, *p* = .002), and WTA was positively related to responsiveness after hours (*b* = 0.54, *SE* = 0.07, *p* < .001). The results support Hypotheses 1, 3, and 5.

Next, we examined indirect effects and obtained 90% confidence intervals (CIs; corresponding to one‐tailed hypothesis tests at the 0.05 level) with a bootstrap method with 5000 replications (Edwards & Lambert, 2007). The results indicate a significant indirect effect of task interdependence on responsiveness after hours through WTA (*b* = 0.13, *SE* = 0.05, 90% CI = [0.064, 0.211]), and a significant indirect effect of WTA on the relationship between dispositional workplace anxiety and responsiveness after hours (*b* = 0.05, *SE* = 0.02, 90% CI = [0.024, 0.091]). The results support Hypotheses 6a and 6b.

**FIGURE 3:**
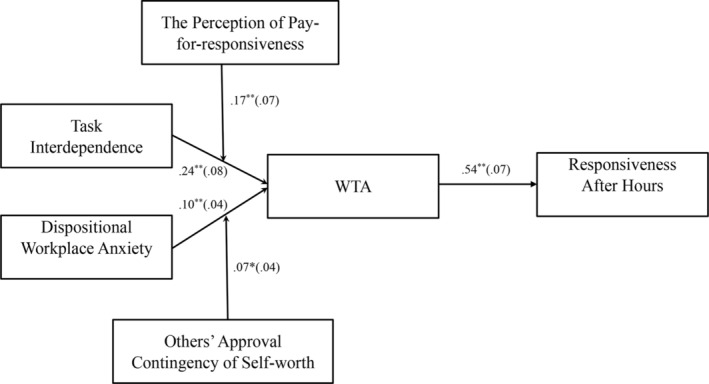
Unstandardized coefficients for the full model (Model 2). WTA = workplace telepressure after hours. Path estimates are unstandardized regression coefficients. Standard errors are in parentheses. The following paths were modeled but excluded from the figure: Task interdependence→Responsiveness after hours, *b* = −0.07, *SE* = 0.07, *p* = .179; Dispositional workplace anxiety→Responsiveness after hours, *b* = −0.07, *SE* = 0.04, *p* = .056; Perception of pay‐for‐responsiveness→WTA, *b* = 0.07, *SE* = 0.04, *p* = .085; Others' approval contingency of self‐worth→WTA, *b* = .04, *SE* = 0.05, *p* = .230. *p < .05, **p < .01, one‐tailed.

**FIGURE 4:**
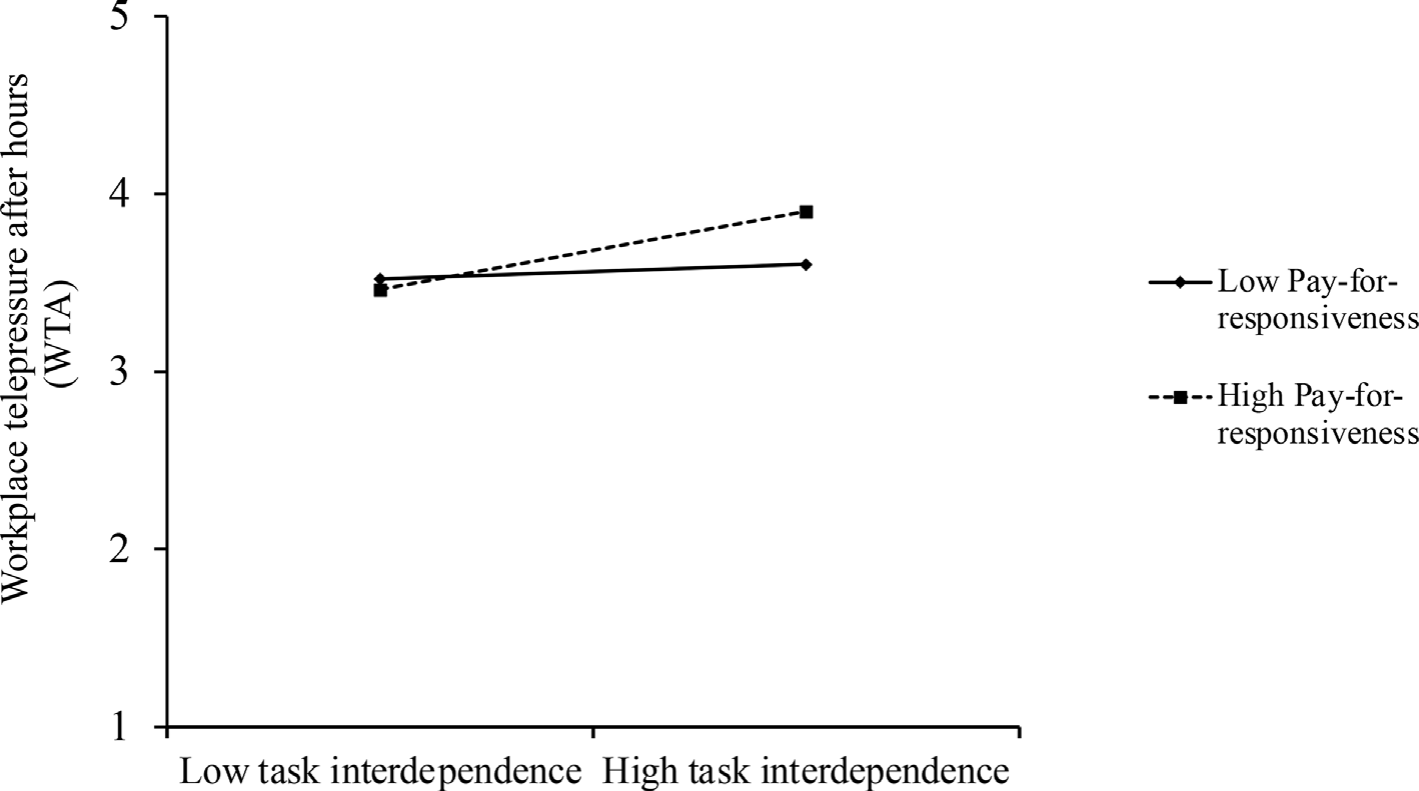
Moderating effect of the perception of pay‐for‐responsiveness on the relationship between task interdependence and WTA. WTA = workplace telepressure after hours.

Next, in the second model, we additionally included the perception of pay‐for‐responsiveness and others' approval contingency of self‐worth as moderators and introduced the interaction effect between the perception of pay‐for‐responsiveness and task interdependence, and the interaction effect between others' approval contingency of self‐worth and dispositional workplace anxiety. This model showed a good fit to the data: *χ* ^2^ (36) = 40.42, CFI = 0.96, TLI = 0.97, RMSEA = 0.02, SRMR = 0.04. The results of the path analysis are displayed in Figure 3. The results show that the perception of pay‐for‐responsiveness significantly moderated the relationship between task interdependence and WTA (*b* = 0.17, *SE* = 0.06, *p* = .009; see Figure 4). A simple slope test analysis showed that the positive effect of task interdependence on WTA was not significant when the perception of pay‐for‐responsiveness was low (–1*SD*; *b* = 0.08, *SE* = 0.08, *p* = .216), but was significant when it was high (+1*SD*; *b* = 0.40, *SE* = 0.11, *p* < .001). Thus, Hypothesis 2 is supported.

**FIGURE 5:**
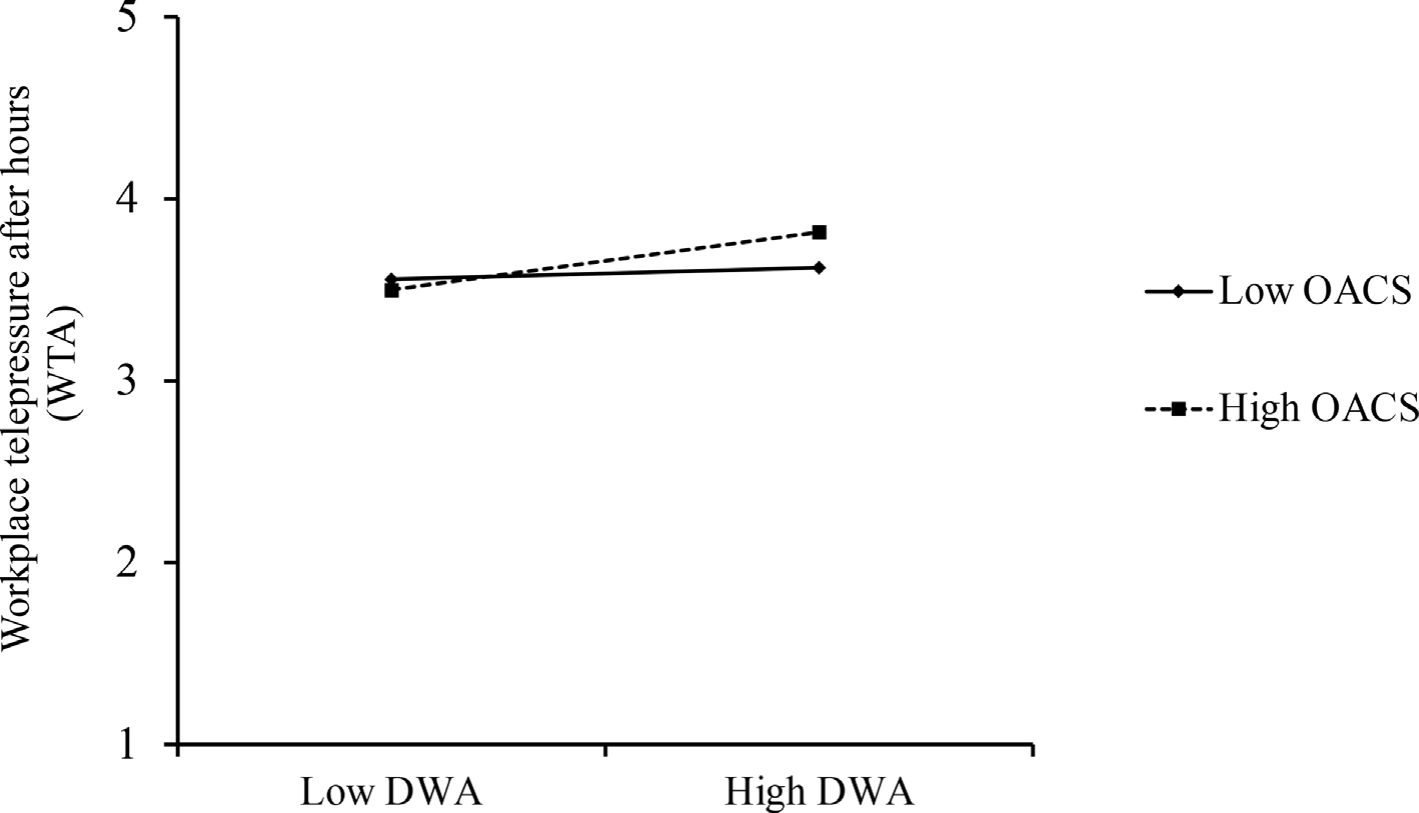
Moderating effect of others' approval contingency of self‐worth on the relationship between dispositional workplace anxiety and WTA. WTA = workplace telepressure after hours; DWA = dispositional workplace anxiety; OACS = others' approval contingency of self‐worth.

Hypothesis 4 proposes that others' approval contingency of self‐worth moderates the relationship between dispositional workplace anxiety and WTA. The results show that others' approval contingency of self‐worth significantly moderated the relationship between dispositional workplace anxiety and WTA (*b* = 0.07, *SE* = 0.04, *p* = .030; see Figure 5). A simple slope test indicated that the relationship between dispositional workplace anxiety and WTA was not significant when others' approval contingency of self‐worth was low (–1*SD*; *b* = 0.02, *SE* = 0.06, *p* = .366). This relationship was significant when others' approval contingency of self‐worth was high (+1 *SD*; *b* = 0.16, *SE* = 0.06, *p* = .002). Therefore, Hypothesis 4 is supported.

Hypotheses 7a and 7b test the indirect effect at different levels of the perception of pay‐for‐responsiveness or others' approval contingency of self‐worth. The results show that the indirect effect of task interdependence on responsiveness after hours through WTA was not significant when the perception of pay‐for‐responsiveness was low (*b* = 0.04, *SE* = 0.05, 90% CI = [−0.039, 0.133]) but was significant when it was high (*b* = 0.22, *SE* = 0.06, 90% CI = [0.120, 0.332]). The difference between the two indirect effects was also significant (*indirect effect difference* = 0.18, *SE* = 0.08, 90% CI [0.053, 0.300]). The indirect effect of dispositional workplace anxiety on responsiveness after hours via WTA was not significant when others' approval contingency of self‐worth was low (*b* = 0.02, *SE* = 0.04, 90% CI = [−0.036, 0.068]) but was significant when others' approval contingency of self‐worth was high (*b* = 0.08, *SE* = 0.04, 90% CI = [0.039, 0.144]). The difference between the two indirect effects was also significant (*indirect effect difference* = 0.07, *SE* = 0.04, 90% CI [0.011, 0.130]). Thus, Hypotheses 7a and 7b are supported.

## DISCUSSION

Drawing on the job demands–resources model, COR theory, and workplace anxiety theory, this study attempts to explore how and when task interdependence and dispositional workplace anxiety affect WTA and how individuals cope with WTA. Using data from 269 full‐time employees, our findings reveal that both task interdependence and dispositional workplace anxiety are positively related to WTA. Employees are more likely to develop the urge to respond quickly to messages when there is a high level of task interdependence and a strong perception of pay‐for‐responsiveness. Furthermore, employees with dispositional workplace anxiety, who value others' evaluations of their self‐worth, generate more WTA. In addition, when employees are in a WTA state, they adopt a strategy of responding promptly to work‐related messages. These results support all seven of our hypotheses.

### Theoretical implications

Our studies make several theoretical contributions. First, our contribution to the telepressure literature lies in the nuanced examination of telepressure during nonworking hours. Such a distinction is very important and moves the telepressure literature forward. During working hours, receiving work‐related messages creates an urge to respond to work‐related messages in a timely manner, which helps to facilitate work completion (Van Laethem et al., 2018). However, when employees return home from work, they are more likely to want to rest to obtain resources to recover (Braukmann et al., 2018). The receipt of work‐related messages during nonworking hours is perceived as an unwelcome interruption, hindering this recovery process (Sonnentag et al., 2018). Integrating the job demands–resources model, COR theory, and workplace anxiety theory, we found that although receiving work‐related information is disruptive to life, employees still have a strong WTA due to job demands (i.e., task interdependence), and personality traits (i.e., dispositional workplace anxiety). Our findings suggest that even the job demands (i.e., task interdependence) and personality traits (i.e., dispositional workplace anxiety) that are not related to ICT could be positively related to WTA. This finding aligns with previous general telepressure research (Barber & Santuzzi, 2015; Grawitch et al., 2018), indicating the broader relevance and consistency of our findings within the telepressure domain.

Second, our study makes a significant contribution by uncovering the boundary conditions that influence the impact of task interdependence and dispositional workplace anxiety on WTA. While existing research focuses on examining the factors affecting telepressure, there has been a notable lack of attention to the boundary conditions on workplace telepressure. For example, Barber and Santuzzi (2015) investigated the positive correlation between job demands related to ICT (i.e., techno‐overload and response norm) and telepressure, as well as the positive relationship between workaholism and telepressure. Similarly, Grawitch et al. (2018) investigated the impacts of workplace demands and individual differences on workplace telepressure. Our study addresses this gap by providing a more nuanced understanding of how and when both job demands and personality traits influence WTA. Specifically, we found that the perception of pay‐for‐responsiveness moderates the relationship between task interdependence and WTA. When employees perceive greater rewards from responding to work‐related messages after hours, there is a stronger relationship between task interdependence and WTA. Moreover, others' approval contingency of self‐worth moderates the relationship between the disposition workplace anxiety and WTA. Employees experience a personal self‐worth manifestation of others' evaluations, and the more employees value others' evaluations of themselves, the stronger their urge to satisfy behaviors that help them achieve others' goals. Thus, employees with dispositional workplace anxiety could feel the urge to prioritize responding to work‐related ICT messages when they place high value on others' approval for their self‐worth. Our findings emphasize the necessity of considering not only the factors influencing telepressure but also the boundary conditions that shape these relationships.

Third, we explored the coping strategies that employees use when they experience WTA. According to the COR theory, we hypothesized that WTA would increase responsiveness after hours. In other words, when individuals are in a WTA state, they are not passive but take the initiative to get tasks done quickly. Prior research revealed that being in WTA for a long period may further deplete an individual's resources, making it possible that they will face a worse outcome (Hu et al., 2019). Responsiveness is an active response to work‐related information that facilitates the perception of work completion and protects individual's resources (Sonnentag et al., 2018). Our results confirmed the positive link between WTA and responsiveness after hours. Moreover, we found that task interdependence and dispositional workplace anxiety had an indirect effect on responsiveness through WTA. These results indicate that both external work demands and personal characteristics can trigger WTA, which in turn leads to responsiveness behavior. Significantly, our study contributes to the finding that when an individual is in a state of WTA, one way to achieve ultimate resource conservation is to quickly respond to work‐related information and complete work tasks. This finding not only enriches the research on outcomes generated by telepressure but also provides new insights into how employees cope with WTA.

### Practical implications

The rapid pace of change in the marketplace puts increasing demands on organizational productivity, and this external demand is passed down through leaders and ultimately to employees. Employees are expected to keep pace by maintaining communication during nonworking hours. It is important to note that while staying online during nonworking hours can improve employee performance and productivity, it can also harm employees' physical and mental health (Golden & Wiens‐Tuers, 2008; Santuzzi & Barber, 2018). As a result, employees may become burned out and less engaged in their work (Barber & Santuzzi, 2015, 2017). To avoid these outcomes, organizations and leaders should be fully aware of the risks of employees dealing with work‐related information during nonworking hours and adopt strategies to reduce the pressure on employees to respond to work‐related information after hours.

First, our findings suggest that task interdependence is associated with WTA, and the perception of pay‐for‐responsiveness moderates this relationship. Given that the nature of a job requires employees to stay in close contact with colleagues or leaders, it is difficult to alter the work environment, but employees can schedule their work hours in advance to avoid unnecessary contact during nonworking hours, for example by negotiating office hours and work content with colleagues in advance and avoiding extra work assignments during the rest of the day. In addition, organizations should set appropriate and reasonable work expectations (Y. Park et al., 2020), reduce rewards for processing work tasks during nonworking hours, and increase rewards for effectively completing work during working hours. In this way, expectations for employees to provide an immediate response to work information can be lowered, thus curtailing WTA.

Second, our findings suggest that disposition workplace anxiety also contributes to the production of WTA, and others' approval contingency of self‐worth plays an important moderating role. Employees can consider decreasing their job expectations to reduce concern about responding to work‐related messages immediately during nonworking hours. Moreover, for employees who worry about others' evaluations of them, not responding to work messages is perceived as not fulfilling others' expectations of their work, which leads to negative results for them and thus prompts more WTA. In relation to this phenomenon, organizations should clarify that employees will not be treated unfairly or evaluated poorly for failing to respond to work‐related messages after hours, and nor will they be given preferential treatment for responding to work‐related ICT messages after hours. Moreover, leaders should also clarify to their employees that their evaluations will not be easily affected by the completion of a task after hours. At the same time, employees should believe in their own competence and personal value and thereby reduce expectations of others' evaluation (Baer et al., 2015). Third, it is important to note that by responding quickly, employees can alleviate the pressure from telepressure and enhance their sense of work completion (Sonnentag et al., 2018). One strategy that employees might consider is actively replying to messages or emails as promptly as possible. This approach not only allows them to complete their work more efficiently but also helps prevent further resource loss.

### Limitations and future directions

The present study has some limitations that need to be addressed in future studies. First, all variables were measured using a self‐report method. Although we took some measures to control the effect of common method bias, such as using anonymous questionnaires and multiple time‐point designs, common method bias issues may remain (Podsakoff et al., 2003). Future studies could consider using multiple sources of data or objective data that can be obtained, such as by tracking and counting the timing and number of emails sent, received, and checked by employees outside of work hours or by measuring the actual work communication behavior of employees during nonworking hours. In addition, the present study used a cross‐sectional research design, which may reduce the presumption of causal relationships between the study variables (Baker et al., 2020). To better illustrate the causal relationships between variables, future studies could collect data via longitudinal follow‐up at multiple time‐points to provide a more rigorous examination of the variables.

Second, some studies have found significant inter‐day fluctuations in employees' workplace telepressure, and that workplace telepressure varies from day to day according to daily workloads and task urgency levels (Becker et al., 2021; Cambier et al., 2019; Y. Park et al., 2020). In this study, WTA was measured only once, and we did not consider the daily change in WTA, so the method lacks ecological validity. Methods such as the experience sampling method (ESM) allow for the collection of events experienced by individuals in their lives multiple times over a relatively short period of time, and use repeated sampling to collect information that is vulnerable to time and personal factors (Bolger & Laurenceau, 2013). Thus, to capture intrapersonal fluctuations in WTA and increase ecological validity, future research could consider using the ESM to examine the impact of work‐related ICT information on employees and explore whether the current theoretical model is still applicable at the inter‐day level.

Third, although the current study focuses on the effects of task interdependence and dispositional workplace anxiety on WTA, it is worth noting that our study and existing studies primarily consider factors that increase employees' WTA, with less attention paid to factors that could decrease it. Future research could further explore the factors that can reduce employees' WTA. From a job resource perspective, mindfulness refers to an individual's tendency to remain attentive and aware of what is happening in the present moment and is an important personal resource (Brown & Ryan, 2003). Employees high in mindfulness are more able to focus on present moment activities, such as recovery or family activities during nonworking hours, and thus may feel less pressure to respond quickly to work‐related information. In addition, in terms of resources in the environment, family‐supportive supervisor behaviors can lead to lowered emotional exhaustion and higher levels of psychological disengagement, and can render employees more inclined to maintain boundaries between work and family (Koch & Binnewies, 2015). When leaders can provide some support or demonstrate exemplary work–family balance behaviors to their employees, this may also reduce the level of WTA felt by employees during nonworking hours. Future research could further consider the above two aspects to examine ways to reduce WTA. Moreover, in our study, we separately examined the impacts of task interdependence and dispositional workplace anxiety on WTA. Investigating whether they will interact to jointly influence WTA could yield valuable insights, further enriching our understanding of the antecedents of WTA. This intersection remains an open question, meriting detailed examination in future studies.

In addition, our results can be interpreted in terms of regulatory focus theory, which may help us to understand the antecedents affecting WTA from another perspective—individuals' motivation. Regulatory focus theory suggests that individuals exhibit specific ways of or tendencies in self‐regulation to achieve goals (Higgins, 1997). The theory distinguishes between two types of regulatory orientations based on the type of need being served: promotion focus, which focuses on achieving personal growth and development; and prevention focus, which focuses on protection from harm (Higgins, 1997). This study reveals two types of boundary conditions for job demands and personal traits that drive employees to remain “always on” during nonworking hours. On the one hand, they may be driven by promotion focus, or striving to meet job demands when it is perceived that they will be rewarded, and focus on the positive outcomes of using nonworking time to respond rapidly to work‐related messages. On the other hand, individuals can be driven by preventive focus, which leads them to perform work when their sense of self‐worth is highly weighted by others' evaluations and to focus on avoiding the negative outcome of not responding to work messages during nonworking time. Future research may go beyond the model of exploring the antecedents of WTA only in terms of environmental and personal factors, examining how motivation drives people to stay “always online” during nonworking hours.

## CONCLUSION

Integrating the job demands–resources model, COR theory, and workplace anxiety theory, our study focused on which factors affect WTA and how employees cope with WTA. Our research shows that task interdependence and dispositional workplace anxiety are positively related to WTA. More importantly, the perception of pay‐for‐responsiveness and others' approval contingency of self‐worth are critical boundary conditions for the influence of task interdependence and dispositional workplace anxiety on WTA, respectively. Moreover, WTA triggers responding to work‐related messages instantly as part of a coping strategy. These findings not only enrich workplace telepressure‐related research but also have theoretical implications for how organizations can manage the workplace expectation to be “always online.”

## CONFLICT OF INTEREST STATEMENT

On behalf of all authors, the corresponding author declares that there is no conflict of interest.

## INFORMED CONSENT

All participants were informed of the research content and signed a consent form before the experiment.

## ETHICS STATEMENT

The experiment was approved by the Research Ethics Board of Beijing Normal University and was performed in accordance with the Declaration of Helsinki.
